# Bioprocessing of Brewers’ Spent Grain Enhances Its Antioxidant Activity: Characterization of Phenolic Compounds and Bioactive Peptides

**DOI:** 10.3389/fmicb.2020.01831

**Published:** 2020-07-31

**Authors:** Michela Verni, Erica Pontonio, Annika Krona, Sera Jacob, Daniela Pinto, Fabio Rinaldi, Vito Verardo, Elixabet Díaz-de-Cerio, Rossana Coda, Carlo Giuseppe Rizzello

**Affiliations:** ^1^Department of Soil, Plant and Food Science, University of Bari Aldo Moro, Bari, Italy; ^2^Research Institute of Sweden, Gothenburg, Sweden; ^3^Giuliani S.p.A., Milan, Italy; ^4^Department of Nutrition and Food Science, University of Granada, Granada, Spain; ^5^Institute of Nutrition and Food Technology “José Mataix”, Biomedical Research Centre, University of Granada, Granada, Spain; ^6^Department of Food and Nutrition Sciences, University of Helsinki, Helsinki, Finland; ^7^Helsinki Institute of Sustainability Science, Helsinki, Finland

**Keywords:** brewers’ spent grain, bioprocessing, phenolic compounds, bioactive peptides, antioxidant activity

## Abstract

Brewers’ spent grain (BSG) is the major by-product of the brewing industry which remain largely unutilized despite its nutritional quality. In this study, the effects of fermentation on BSG antioxidant potential were analyzed. A biotechnological protocol including the use of xylanase followed by fermentation with *Lactiplantibacillus plantarum (Lactobacillus plantarum)* PU1, PRO17, and H46 was used. Bioprocessed BSG exhibited enhanced antioxidant potential, characterized by high radical scavenging activity, long-term inhibition of linoleic acid oxidation and protective effect toward oxidative stress on human keratinocytes NCTC 2544. Immunolabelling and confocal laser microscopy showed that xylanase caused an extensive cell wall arabinoxylan disruption, contributing to the release of bound phenols molecules, thus available to further conversion through lactic acid bacteria metabolism. To clarify the role of fermentation on the antioxidant BSG potential, phenols were selectively extracted and characterized through HPLC-MS techniques. Novel antioxidant peptides were purified and identified in the most active bioprocessed BSG.

## Introduction

Brewers’ spent grain (BSG), the major by-product of the beer-brewing industry, consists of the seed coat–pericarp–husk layers covering the original barley (*Hordeum vulgare*) grain (Lynch et al., 2016), and of insoluble residues deriving from other additional ingredients, such as raw or malted cereals, like maize (*Zea mays*) or wheat (*Triticum durum* and *Triticum aestivum*). Briefly, after extraction of the soluble compounds from the barley into the mash, the BSG is separated from the wort (liquid), which is then boiled with hop and fermented. BSG represents a very abundant by-product with an average annual global production of approximately 39 million tons, 10% of which produced in Europe only (Lynch et al., 2016).

Brewers’ spent grain is essentially made of fiber (mainly hemicellulose and cellulose) which constitute half of its composition on dry basis; other important constituents are protein (up to 30%) and phenols. Arabinoxylans (AX), the main hemicellulose constituent, have a backbone of xylose residues, which can be substituted with arabinose residues (Lynch et al., 2016). BSG arabinoxylans include a significant water-extractable fraction and during enzymatic digestion they can release xylooligosaccharides with varying degree of polymerization (Wang et al., 2010).

Brewers’ spent grain is a valuable source of phenolics, whose antioxidant, antiradical, but also anti-carcinogenic and anti-apoptotic properties, are widely recognized (Lynch et al., 2016). BSG phenolics are mainly contained in the barley husk, accumulated in cell walls and esterified to sugar residues, conditions that decrease their antioxidant activity since availability of free hydroxyl group is essential to stabilize free radicals (Bhanja et al., 2009). The major part of phenolic compounds in BSG are hydroxycinnamic acids, of which ferulic acid is the most abundant and in strong correlation with cereal antioxidant capacity. However, since ferulic acid is bound to the ligninocellulosic structure of BSG, it has low bioaccessibility. Bioprocessing can improve the bioaccessibility of phenolic compounds from fiber-rich cereal matrix. In a previous study, a variety of hydrolytic enzymes, mainly xylanase, β-glucanase, α-amylase, cellulase, and ferulic acid esterase coupled with yeast fermentation was used to treat wheat bran with the aim of improving the bioaccessibility of phenolic compounds in bran-containing breads. This treatment led to an increase of the bioaccessibility of ferulic acid and other phenolic acids of bran *in vitro* (Anson et al., 2009).

In addition to the potential health benefits, when added to food, antioxidants control rancidity development, maintaining the nutritional quality and shelf-life of products. Nowadays, there is still a growing interest in finding natural sources of antioxidants to effectively replace synthetic ones, which have been related to toxic and carcinogenic effects (Bouayed and Bohn, 2010).

Brewers’ spent grain protein component is also of interest, thanks to the lysine abundance in comparison with other cereal-derived matrices, and for the antioxidant, anti-inflammatory and immunomodulatory potential of the proteolysis derivatives, previously investigated trough *in vitro* and *ex vivo* assays (Lynch et al., 2016). Additionally, BSG can also be a source of antioxidant peptides as previously shown for several cereal flours, including whole wheat. These bioactive peptides, released through fermentation with lactic acid bacteria (LAB), showed protection of cells from oxidation (*ex vivo*) as well as inhibition of linoleic acid oxidation (Verni et al., 2019).

Despite the high nutritional and functional potential, most of the BSG produced is used for feed or as substrate to produce bioethanol, lactic acid, xylitol and enzymes (Nigam, 2017). Yet, BSG use as food ingredient is more limited due to its complex structure and consequent poor technological performance, which requires treatments able to reduce the negative impact on texture and sensory properties of the final food product (Lynch et al., 2016). Among these, pre-treatments with xylanase were effective in improving specific volume and texture of breads supplemented with BSG and increased the amount of soluble fibers (Ktenioudaki et al., 2015). Similarly, fermentation, largely recognized as a technology able to improve sensory and technological properties of fiber-rich cereal by-products, was proven helpful to enhance nutritional and functional properties of BSG in different bread and beverages applications (Gupta et al., 2013).

The focus of this study was to understand the effect of fermentation on the antioxidant properties of BSG. To enable the release or synthesis of antioxidant compounds in BSG, a biotechnological process based on fermentation with selected LAB and treatment with a commercial xylanase was developed.

The effects of bioprocessing on the antioxidant activity were investigated through an integrated multi-step approach including *in vitro* and *ex vivo* assays, study of BSG microstructure, and characterization of phenolic compounds and peptide profile.

## Materials and Methods

### Microorganisms

Thirty-three LAB strains, *Lactiplantibacillus plantarum* (formerly classified as *Lactobacillus plantarum*) LB1, C2, C48, H46, H48, T6B4, T0A10, T6C16, 18S9, MRS1, 1A7, PU1, PRO17, *Furfurilactobacillus rossiae* (formerly classified as *Lactobacillus rossiae*) LB5, T0A16, *Levilactobacillus brevis* (formerly classified as *Lactobacillus brevis*) MRS4, AM7, *Pediococcus pentosaceus* H11, T1A13, I76, I214, I02, I014, F01, OA1, S3N3, BAR4, *Pediococcus acidilactici* 10MM0, *Pediococcus* sp. I56, *Leuconostoc mesenteroides* 12MM1, I57, *Weissella confusa* KAS3, and NEY6, all belonging to the Culture Collection of the Department of Soil, Plant and Food Sciences (University of Bari, Italy) were used to ferment BSG. All the strains were previously used as starters for fermentation and characterized for pro-technological properties (growth and acidification performances, as well as the ability to increase antioxidant activity) as expressed in their own food isolation matrix (wheat, wheat germ quinoa, hop, hemp, faba bean, chickpea, carrots) (Di Cagno et al., 2008; Rizzello et al., 2010, 2016; Pontonio et al., 2015; Mamhoud et al., 2016; Nionelli et al., 2018).

The strains were routinely propagated on De Man, Rogosa, and Sharpe (MRS) (Oxoid, Basingstoke, Hampshire, United Kingdom) at 30°C. Before inoculation they were cultivated until the late exponential phase of growth was reached (*ca*. 10 h), harvested by centrifugation at 9000 × *g* for 10 min at 4°C, washed twice in 50 mM sterile phosphate buffer (4°C, pH 7.0), resuspended in sterile distilled water and used to inoculate BSG.

### Raw Material and Enzymes

The BSG employed in this study, kindly provided by Peroni brewery (Bari, Italy), is obtained from the production of a lager beer brewed with barley malt (70%) and maize (*Z. mays*) (30%) and do not contain spent yeast. Before any experiment, BSG was grinded with a laboratory mill Ika-Werke M20 (GMBH, and Co. KG, Staufen, Germany). After milling, the BSG particle size distribution was: 250–500 μm (30%), 500–750 μm (60%), 750–1000 μm (10%). BSG proximal composition was: moisture, 80 ± 0.07%; protein, 21.11 ± 0.19% of dry matter (d.m.); fat, 10.89 ± 0.01% of d.m.; cellulose, 22.51 ± 0.68% of d.m.; hemicellulose, 24.81 ± 0.97% of d.m, lignin, 15.33 ± 0.28% of d.m; ashes, 5.12 ± 0.05% of d.m.

The commercial hydrolytic enzyme, Depol^TM^ 761P (Biocatalysts, Chicago, IL, United States), a preparation derived from *Bacillus subtilis* having xylanase activity (14,670 nkat/g), was used either individually or in combination with three selected strains for BSG fermentation.

### BSG Bioprocessing

#### Starter Selection

Lactic acid bacteria strains were singly inoculated (initial cell density ca. 7.5 cfu/g) in BSG homogenized with water at a 60:40 ratio and incubated at 30°C for 24 h. Native BSG and BSG incubated without the inoculum were used as control (Ct t0 and Ct t24). Fermentation was monitored by measuring, before and after incubation, pH and enumerating presumptive LAB using MRS (Oxoid, Basingstoke, Hampshire, United Kingdom) agar medium, supplemented with cycloheximide (0.1 g/L). Plates were incubated in anaerobiosis condition (AnaeroGen and AnaeroJar, Oxoid) at 30°C for 48 h. Yeasts, molds and total *Enterobacteria* were respectively enumerated on: Sabouraud Dextrose Agar (Oxoid) supplemented with chloramphenicol (0.1 g/L) at 25°C for 48 h; Potato Dextrose Agar (Oxoid) at 25°C for 48 h, and Violet Red Bile Glucose Agar (Oxoid) at 37°C for 24 h.

To select the strains able to induce the highest increase of the antioxidant activity, fermented BSG was subjected to methanolic and aqueous extraction, and extracts were analyzed for the radical scavenging activity, total phenols, peptide, and free amino acids concentration as described in section “Antioxidant Activity.”

#### Set-up of the Bioprocessing Protocol

The enzymatic treatment with Depol (100 nkat/g dough) was performed contextually to fermentation with the strains *L. plantarum* PU1, H46, and PRO17 (initial cell density ca. 7.5 cfu/g) at 30°C for 24 h (EF30), or before fermentation, at 50°C for 5 h (E50 + F30) (Severini et al., 2015). Samples of BSG not inoculated with the LAB strains but added with xylanase and incubated in the conditions used for fermentation, were also prepared and used as control (eBSG). Antioxidant activity was evaluated on untreated, raw BSG (rBSG), eBSG, BSG treated with xylanase and fermented with *L. plantarum* PU1 (eBSG fPU1), H46 (eBSG fH46), and PRO17 (eBSG fPRO17). The list of treatments and codes is reported in Table 1.

**TABLE 1:** List of treatments and samples codes.

**Code**	**Description**
rBSG	Untreated, raw BSG
EF30: Bioprocessing protocol including addition of xylanase and simultaneous fermentation at 30°C for 24 h	
*eBSG*	BSG added with xylanase, not inoculated and incubated at 30°C for 24 h
*eBSG fH46*	BSG added with xylanase, inoculated with *L. plantarum* H46 and incubated at 30°C for 24 h
*eBSG fPU1*	BSG added with xylanase inoculated with *L. plantarum* PU1 and incubated at 30°C for 24 h
*eBSG fPRO17*	BSG added with xylanase inoculated with *L. plantarum* PRO17 and incubated at 30°C for 24 h
E50 + F30: Bioprocessing protocol including sequential treatment with xylanase at 50°C for 5 h and fermentation at 30°C for 24 h	
*eBSG*	BSG treated with xylanase at 50°C for 5 h, and further incubated at 30°C for 24 h (not inoculated)
*eBSG fH46*	BSG treated with xylanase at 50°C for 5 h, then inoculated with *L. plantarum* H46 and fermented at 30°C for 24 h.
*eBSG fPU1*	BSG treated with xylanase at 50°C for 5 h, then inoculated with *L. plantarum* PU1 and fermented at 30°C for 24 h.
*eBSG fPRO17*	BSG treated with xylanase at 50°C for 5 h, then inoculated with *L. plantarum* PRO17 and fermented at 30°C for 24 h.

Fermentation was monitored by measuring, before and after incubation, pH, enumerating presumptive LAB, yeasts, molds, and total *Enterobacteria* as described above.

### Antioxidant Activity

#### Antioxidant Activity in Methanolic Extracts

Five grams of each sample were mixed with 50 mL of 80% methanol to get methanolic extracts (ME). The mixture was purged with nitrogen stream for 30 min, under stirring condition, and centrifuged at 4600 × *g* for 20 min. ME were further purged with nitrogen stream and stored at ca. 4°C before analysis. The 2,2-diphenyl-1-picrylhydrazyl (DPPH) radical scavenging activity was determined on ME as described by Yu et al. (2003). The scavenging activity was expressed as follows: DPPH scavenging activity (%) = [(blank absorbance−sample absorbance)/blank absorbance] × 100. The value of absorbance was compared with 75 ppm butylated hydroxytoluene (BHT), which was used as the antioxidant reference. The analysis of total phenols in ME was performed according to the method of Slinkard and Singleton (1997) using gallic acid as standard.

#### Antioxidant Activity in Water/Salt Soluble Extracts (WSE)

Since polyphenols are not the only compounds capable of improving antioxidant properties, water/salt soluble extract (WSE) from raw or bioprocessed BSG were prepared according to the method originally described by Osborne and modified by Weiss et al. (1993). In details, 25 g of BSG slurry were suspended in 12 mL of 50 mM Tris–HCl (pH 8.8), held at 4°C for 1 h, in stirring conditions, and centrifuged at 20,000 *g* for 20 min. The supernatant was used for analyses. The radical cation [2,2′-azino-di-(3-ethylbenzthiazoline sulfonate)] (ABTS+) scavenging capacity of the WSE was measured using the Antioxidant Assay Kit CSO790 (Sigma Chemical Co.), following the manufacturer’s instruction. Trolox (6-hydroxy 2,4,7,8-tetramethylchroman-2-carboxylic acid) was used as standard. The scavenging activity was expressed as Trolox equivalent.

Water/salt soluble extract were then used to analyze peptides, and total free amino acids (TFAA). Peptides concentration was determined by the *o*-phtaldialdehyde (OPA) method as described by Church et al. (1983). TFAA were analyzed by a Biochrom30 series Amino Acid Analyzer (Biochrom Ltd., Cambridge Science Park, United Kingdom) with a Na-cation exchange column (20 cm × 0.46 cm internal diameter as reported in Rizzello et al., 2010).

#### Inhibition of Linoleic Acid Peroxidation

One milligram of freeze-dried WSE and ME of each sample was suspended in 1.0 mL of 0.1 M phosphate buffer (pH 7.0) and added to 1 mL of linoleic acid (50 mM), previously dissolved on ethanol (99.5%). After incubation, at 60°C in the dark for 8 days, in a glass test tube tightly sealed with silicone rubber cap, linoleic acid degree of oxidation was determined by measuring the values of ferric thiocyanate according to the method described by Mitsuda (1966). One hundred microliters of the above sample were mixed with 4.7 mL of 75% (v/v) ethanol, 0.1 mL of 30% (w/v) ammonium thiocyanate, and 0.1 mL of 0.02 M ferrous chloride, dissolved in 1 M HCl. After 3 min, the degree of color development, representing the oxidation of linoleic acid, was measured spectrophotometrically at 500 nm. BHT (1 mg/mL) was also assayed as antioxidant references. A reference sample (without the addition of antioxidants) was included in the assay as negative control.

### Microstructure Characterization and Dietary Fibers Analysis

#### Sample Preparation for Microscopy

Brewers’ spent grain treated as described in section “Set-up of the Bioprocessing Protocol” were frozen in liquid nitrogen, embedded in PELCO Cryo-Embedding Compound (Ted Pella inc., Redding, CA, Untied States) and cut into 10 μm thick sections in a Leica CM 1900. Sections were applied to polysine coated microscopy slides.

#### Bright Field Microscopy

Sections were stained with light green to visualize starch and proteins. The stained sections were examined using a Nikon microphot-FXA microscope and micrographs captured with a DFK33UX264 camera (The Imaging Source Europe GmbH, Bremen, Germany) and processed with the software NIS-Elements D (Nikon Instruments Europe, Amsterdam, Netherlands).

#### Immunolocalization of AX by Confocal Laser Microscopy

For immunolabelling, sections were fixated for 30 min in 4% paraformaldehyde in PBS buffer (pH 7.4) and rinsed with PBS. The sections were then pre-incubated 40 min in PBS buffer with 2.5% BSA before applying the primary antibody, LM11 (Plant Probes, Leeds, United Kingdom) diluted 1:50 in PBS containing 0.5% BSA, for 2 h. Negative controls were made by replacing the primary antibody solution with PBS containing 0.5% BSA. After incubation, the sections were rinsed thoroughly with PBS and then incubated for 2 h in the dark with fluorescently labeled secondary antibody Alexa Fluor^®^ 647 (Invitrogen, Carlsbad, CA, United States). All incubations were performed in moisturized chambers at room temperature. Sections were then rinsed with PBS and water and mounted with ProLong^®^Diamond (Invitrogen, Carlsbad, CA, Untied States) anti-fading reagent.

Micrographs were acquired using a confocal laser microscopy (CLSM; Leica TCS SP5, Heidelberg, Germany) A 488 nm argone laser and a 633 nm HeNe laser with a HCX PL APO lambda blue 20.0 × 0.70 IMM UV objective, zoom 1× and 3×. Emissions were collected at 500–550 and 650–700 nm. Image format 1024 × 1024 pixels, eight lines average.

#### Dietary Fibers Analysis

To further confirm the results obtained from microscopy analysis, insoluble and soluble dietary fibers content was determined in raw and bioprocessed BSG according to the official AOAC International (2007).

### *Ex vivo* Assays on Human Keratinocytes

The human keratinocyte cell line NCTC 2544 was obtained from the National Institute for Cancer Research of Genoa, Italy. Cells were cultivated in RPMI-1640 medium supplemented with 10% fetal bovine serum (FBS), 2 mM l-glutamine, 1% penicillin (100 U/mL)/streptomycin (100 U/mL) and 0.1% of gentamicin. NCTC 2544 cells were incubated in 25 cm^2^ surface culture flasks at 37°C with 5% CO_2_ (Sanchez et al., 2004). When ca. 80% of confluence was reached, cells were harvested with trypsin/EDTA and seeded at a density of 5 × 10^4^ cells per well into 96 well plates for 3-(4,5-dimethylthiazol-2-yl)-2,5-diphenyltetrazolium bromide proliferation (MTT) assay, and oxidative stress tests. Medium and all chemicals were purchased from Sigma Aldrich (Italy).

3-(4,5-dimethylthiazol-2-yl)-2,5-diphenyltetrazolium bromide proliferation assay was used for the determination of the viability of H_2_O_2_-stressed NCTC 2544 cells (Coda et al., 2012). Cells were incubated with raw and bioprocessed BSG WSE for 16 h. The concentrations were 0.1, 0.5, and 1 mg/mL; α-tocopherol (250, 500, and 1000 μg/mL) was used as the positive control. At the end of incubation, MTT assay was performed (Mosmann, 1983). Data were expressed as the percentage of viable cells compared to negative control. Each experiment was carried out in triplicate.

### Characterization of the Phenolic Profile

#### Extraction of Phenolic Compounds and Qualitative and Quantitative Analysis by UPLC-PDA-ESI-QTOF

Free and bound phenolics were extracted as described in Verardo et al. (2011). The analysis of BSG free and bound polyphenols was carried out with the use of an ACQUITY Ultra Performance LC system equipped with photodiode array detector with a binary solvent manager (Waters Corporation, Milford, MA, United States) series with a mass detector Q/TOF micro mass spectrometer (Waters) equipped with an electrospray ionization (ESI) source operating in negative mode at the following conditions: capillary voltage, 2300 kV; source temperature, 100°C; cone gas flow, 40 L/Hr; desolvatation temperature, 500°C; desolvatation gas flow, 11,000 L/h; and scan range, m/z 50–1500. Separations of individual polyphenols were carried out using an ACQUITY UPLC BEH Shield RP18 column (1.7 μm, 2.1 mm × 100 mm; Waters Corporation, Milford, MA, United States) at 40°C. The elution gradient was carried out using water containing 1% acetic acid (A) and acetonitrile (B), and applied as follows: 0 min, 1% B; 2.3 min, 1% B; 4.4 min, 7% B; 8.1 min, 14% B; 12.2 min, 24% B; 16 min, 40% B; 18.3 min, 100% B, 21 min, 100% B; 22.4 min, 1% B; 25 min, 1% B. The sample volume injected was 2 μL and the flow rate used was 0.6 mL/min. The compounds were monitored at 280 nm. Integration and data elaboration were performed using MassLynx 4.1 software (Waters Corporation, United States). For the quantification of phenolic compounds, solutions of ferulic acid, chlorogenic acid, catechin, and quercetin in methanol were prepared and used as standard.

#### Extraction and Quantification of Proanthocyanidins

Proanthocyanidins were extracted as described by Verardo et al. (2015) and analyzed by HPLC-FLD analysis, performed by an Agilent 1200 Series (Agilent Technologies, Santa Clara, CA, United States), equipped with a Luna Hilic column (150 × 2.0 mm; 3 μm) and a fluorescence detector with an excitation wavelength of 230 nm and an emission wavelength of 321 nm. The separation was performed with 98% acetonitrile and 2% acetic acid (A) and 95% methanol, 3% water, and 2% acetic acid (B) as described by Hollands et al. (2017).

### Characterization of Peptide Profiles

#### Purification of Antioxidant Peptides

Water/salt soluble extracts obtained from BSG treated with xylanase at 50°C for 5 h and further fermented with *L. plantarum* PU1, which showed the highest radical scavenging activity, was first fractionated by ultra-filtration (Ultrafree-MC centrifugal filter units, Millipore) using membrane sizes of 50, 30, 10, and 3 kDa cut-off, following the manufacturer’s instructions. Fractions were tested for the antioxidant activity on DPPH radical. The WSE fraction with a molecular mass of <3 kDa was further automatically fractionated (2 mL per fraction, 32 fractions for each run) by reversed-phase fast performance liquid chromatography (RP-FPLC), using a Resource RPC column and an ÄKTA FPLC equipment, with the UV detector operating at 214 nm (GE Healthcare Bio-Sciences AB, Uppsala, Sweden). Gradient elution was performed at a flow rate of 1 mL/min using a mobile phase composed of water and acetonitrile (CH_3_CN), containing 0.05% TFA. The concentration of CH_3_CN was increased linearly from 5 to 46% between 16 and 62 min, and from 46 to 100% between 62 and 72 min. Solvents were removed from collected fractions by freeze drying. Fractions were re-dissolved in sterile water, assayed for the antioxidant activity on DPPH radical and the peptide content by the OPA method as described above (Rizzello et al., 2010).

#### Proteolysis and Heat Stability

Water/salt soluble extracts and purified fractions, which showed the highest antioxidant activities, were subjected to sequential protein hydrolysis by digestive enzymes (pepsin, pancreatin, and trypsin) according to the method described by Pasini et al. (2001). Digested samples were heated for 5 min at 100°C and centrifuged at 12,000 × *g* for 20 min to recover the supernatants. After treatments, samples were subjected the scavenging activity on radical DPPH as described above.

#### Identification of Antioxidant Peptides

The peptides contained in the WSE fractions with the highest radical-scavenging activity were subjected to identification. The identification was carried out by nano-Liquid Chromatography-Electrospray Ionization-Mass Spectra/Mass Spectra (nano-LC-ESI-MS/MS), using a Finnigan LCQ Deca XP Max ion trap mass spectrometer (ThermoElectron) through the nano-ESI interface. According to manufacturer’s instrument settings for nano-LC-ESI-MSMS analyses, MS spectra were automatically taken by Xcalibur software (ThermoElectron), in positive ion mode. MS/MS spectra were processed using the software BioWorks 3.2 (ThermoElectron) generating peak lists suitable for database searches. Peptides were identified using MS/MS ion search of Mascot search engine (Matrix Science, London, United Kingdom) and NCBInr protein database (National Centre for Biotechnology Information, Bethesda, MD, United States). For identification of peptides the following parameters were considered: enzyme: “none”; instrument type: “ESI-trap”; peptide mass tolerance: ±0.1% and fragment mass tolerance: ±0.5 Da. Results from peptide identification were subjected to a manual evaluation, as described by Chen et al. (2005), and the validated peptide sequences explained all the major peaks in the MS/MS spectrum.

### Statistical Analysis

All the microbiological and chemical analysis were carried out in triplicate for each batch of BSG. Data were subjected to one-way ANOVA; pair-comparison of treatment means was achieved by Tukey’s procedure at *P* < 0.05, using the statistical software Statistica 8.0 (StatSoft Inc., Tulsa, OK, United States). Data of the *ex vivo* assays were analyzed with a statistical software: GraphPad Prism v7.00 (GraphPad Software Inc.). Student’s *t*-test with was Mann–Whitney correction was used. *P*-values equal to or less than 0.05 were considered significant.

## Results

### Starter Selection

Before incubation BSG had a pH of 5.87 ± 0.08 which significantly decreased after 24 h of spontaneous fermentation at 30°C (5.01 ± 0.05). After fermentation, the pH of the inoculated samples ranged from 3.87 ± 0.03 to 4.42 ± 0.04. The lowest variation was observed when BSG was fermented with *W. confusa* NEY6. On the contrary, *L. plantarum* H48, PU1, T6C16, T0A10, PRO17, *P. pentosaceus* OA1, and *W. confusa* KAS3 caused the highest pH decrease, reaching values lower than 4 at the end of fermentation.

Cell density of presumptive LAB in rBSG was lower than 2 log cfu/g, while yeasts and total *Enterobacteriaceae* were 4.76 ± 0.03, and 3.58 ± 0.03 log10 cfu/g, respectively. Molds were not found (<10 cfu/1 g). When BSG was inoculated (initial cell density ca. 7.5 log10 cfu/g) an increase of *ca*. 2 log cycles of presumptive LAB cell density was observed (final values ranged from 9.36 to 9.67 log10 cfu/g). The highest final cell density was observed for *L. plantarum* H48, 1A7, and PRO17. At the end of fermentation, yeasts and *Enterobacteriaceae* cell density was lower than 4.12 ± 0.02 and 2.10 ± 0.03 log10 cfu/g, respectively, in all the BSG samples.

The main criterion for LAB selection was the ability of fermented BSG extracts to scavenge DPPH and ABTS radicals, compared to the untreated BSG. The scavenging activity on DPPH for rBSG was 42.5 ± 2.3%. Overall, activity increments up to 7% were observed. The highest increase (*P* < 0.05) was observed for *L. plantarum* PU1 and H46 with antioxidant activity of 48 ± 0.96 and 49 ± 0.78%, respectively. Significant (*P* < 0.05) increases of the ABTS scavenging activity were also found during fermentation. BSG fermented with *L. plantarum* PRO17, *P. pentosaceus* I76 and I214 showed an increase (*P* < 0.05) exceeding 0.2 mM Trolox equivalent, more than 33% higher than BSG before fermentation (0.17 ± 0.05 mM Trolox equivalent).

The total phenol content of BSG methanol extracts was determined using the Folin–Ciocalteu method. Before incubation rBSG had total phenol content 2.09 ± 0.16 mmol/100 g. Except for the spontaneously fermented control (Ct t24), in which a decrease (*P* < 0.05) was observed, for all fermented BSG, total phenol content remained stable or increased. In BSG fermented with *L. plantarum* LB1, 18S9, MRS1, PRO17, *F. rossiae* LB5, T0A16, *Lv. brevis* MRS4, *P. pentosaceus* BAR4, OA1, S3N3 an increase ranging from 10 to 20% was observed. Fermentation with *L. plantarum* PU1, *L. mesenteroides* 12MM1, I57, *P. pentosaceus* I56, I76 and F01 allowed an increase higher than 30%, reaching values up to 2.71 mmol/100 g.

Peptides concentration before fermentation was 74.65 ± 2.25 mg/g (d.m.) and no changes were observed after spontaneous fermentation. When fermented with LAB, 12 out of 33 strains caused an increase of peptide content up to 20%, reaching 98 mg/g when fermented with *L. mesenteroides* 12MM1. Amino acids content was from two to ninefold higher than that of rBSG (215 ± 10.87 mg/kg on d.m.) in all the fermented BSG samples.

**FIGURE 1:**
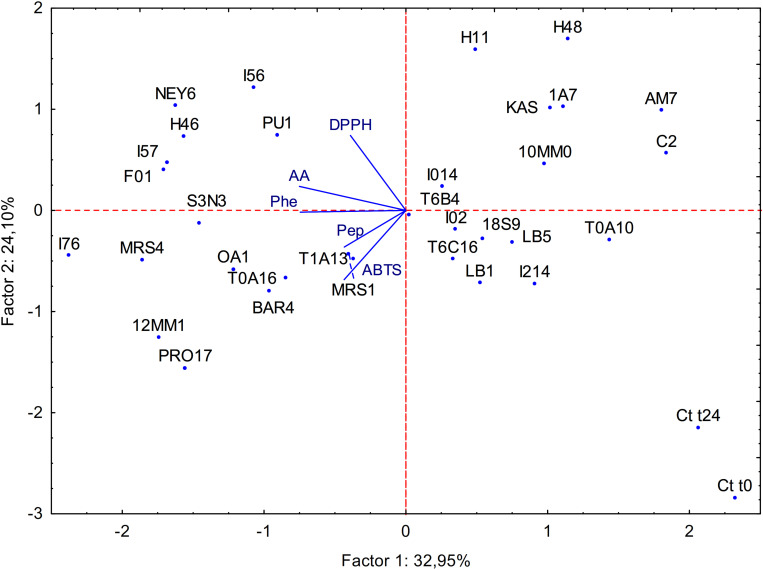
Principal component analysis (PCA) of the biochemical characteristics of raw BSG (Ct t0), spontaneously fermented BSG (Ct t24) and BSG fermented with selected acid bacteria (name of the strains are reported in section “Microorganisms”). ABTS, antioxidant activity on ABTS; Pep, peptide concentration; AA, amino acid concentration; Phe, phenolic compounds content; DPPH, antioxidant activity on DPPH.

All the data obtained from the biochemical characterization of raw and fermented BSG were subjected to principal component analysis (PCA) as shown in Figure 1. Factor 1 and 2 represented 32.95 and 24.10% of the variance, respectively. Untreated BSG and BSG incubated without LAB clearly separated from the fermented sample; Factor 1 separated fermented BSG having lower content in peptides and amino acids from those with higher content.

### Bioprocessing

**FIGURE 2:**
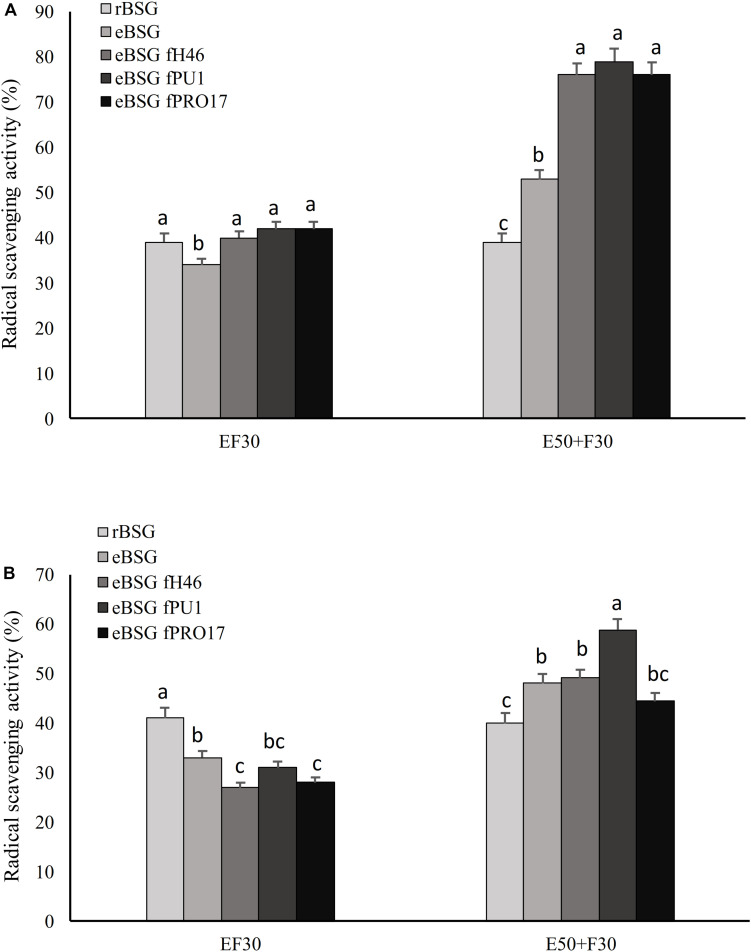
Scavenging activity on DPPH radical of the water/salt soluble **(A)** and methanol extracts **(B)** of raw BSG (rBSG); BSG treated with xylanase and not inoculated (eBSG); BSG treated with xylanase and fermented with *L. plantarum* PU1 (eBSG fPU1), H46 (eBSG fH46), and PRO17 (eBSG fPRO17). The enzymatic treatment was performed contextually to fermentation at 30°C (EF30) for 24 h, or before fermentation at 50°C for 5 h (E50 + F30). Data are the means from three independent experiments. Bars represent standard deviation. ^a–c^Columns with different superscript letters, within the same protocol, differ significantly (*P* < 0.05).

*Lactiplantibacillus plantarum* PU1, H46, and PRO17 were used in combined bioprocessing with Depol 761P. The use of the enzyme simultaneously with LAB did not enhance the scavenging activity on DPPH compared to BSG fermented without enzyme (Figure 2).

On the contrary, the enzymatic treatment before fermentation at 50°C for 5 h positively influenced the antioxidant properties, increasing the radical scavenging activity up to 18% in ME and 40% in WSE. Compared to rBSG, the xylanase treatment carried out without the inoculum of the selected LAB (eBSG) led to a slight but significant decrease of the antioxidant activity when incubation was carried out at 30°C for 24 h (EF30), or to a significantly lower increase than those observed for eBSF fH46, eBSG fPU1, eBSG fPRO17 when the pre-incubation at 50°C for 5 h was carried out (Figure 2). In both cases, cell density of LAB ranged from 5.46 ± 0.03 to 5.71 ± 0.02 log10 cfu/g, while yeasts and *Enterobacteriaceae* were 5.42 ± 0.02 and 4.50 ± 0.03, and 2.42 ± 0.03 and 4.20 ± 0.03 log10 cfu/g, respectively in EF30 and E50 + F30 eBSG. Based on these results, the sequential use of xylanase and LAB fermentation E50 + F30 was selected as the bioprocessing option and used for further experiments.

### Linoleic Acid Peroxidation

**FIGURE 3:**
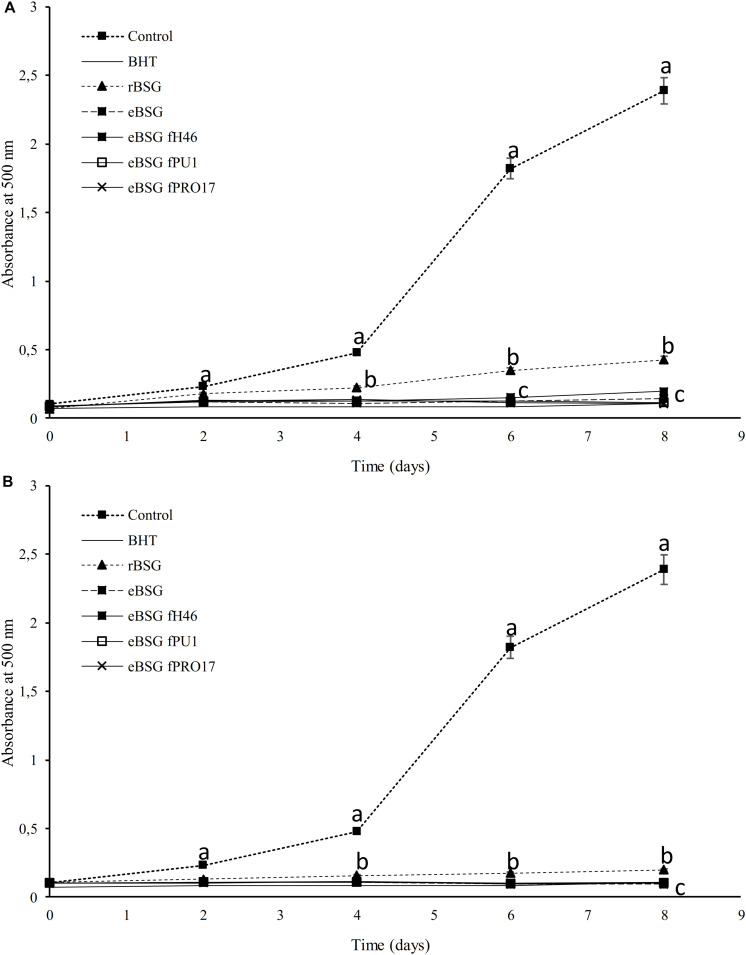
Lipid peroxidation inhibitory activity of the WSE **(A)** and ME **(B)** in raw BSG (rBSG); BSG treated with xylanase and not inoculated (eBSG); BSG treated with xylanase and fermented with *L. plantarum* PU1 (eBSG fPU1), H46 (eBSG fH46), and PRO17 (eBSG fPRO17). The activity was measured under linoleic acid oxidation system for 8 days. BHT (1 mg/mL) was used as the positive control. The reaction mixture without the addition of the antioxidant was considered as a negative control. Data are the means from three independent experiments. Bars represent standard deviation. ^a–c^Points with different superscript letters differ significantly (*P* < 0.05).

Extracts from raw and treated BSG were tested for the ability to inhibit lipid peroxidation. During linoleic acid oxidation, peroxides oxidize Fe^2+^ to Fe^3+^. The Fe^2+^ ions form a complex with thiocyanate, which has a maximum absorbance at 500 nm. In this method, the concentration of peroxides decreases as the antioxidant activity increases (Omwamba and Hu, 2010). Compared to the reference (reaction mixture without antioxidants), the presence of all extracts inhibited linoleic acid autoxidation (Figure 3). The oxidation of linoleic acid was markedly inhibited by the addition of WSE and ME from bioprocessed BSG, comparable to that of BHT. There were no differences (*P* < 0.05) among bioprocessed samples whereas the WSE and ME of rBSG had a significant lower effect on linoleic acid inhibition.

### Microstructure Characterization and Dietary Fibers and Analysis

#### Morphology Characterization of Bioprocessed BSG

**FIGURE 4:**
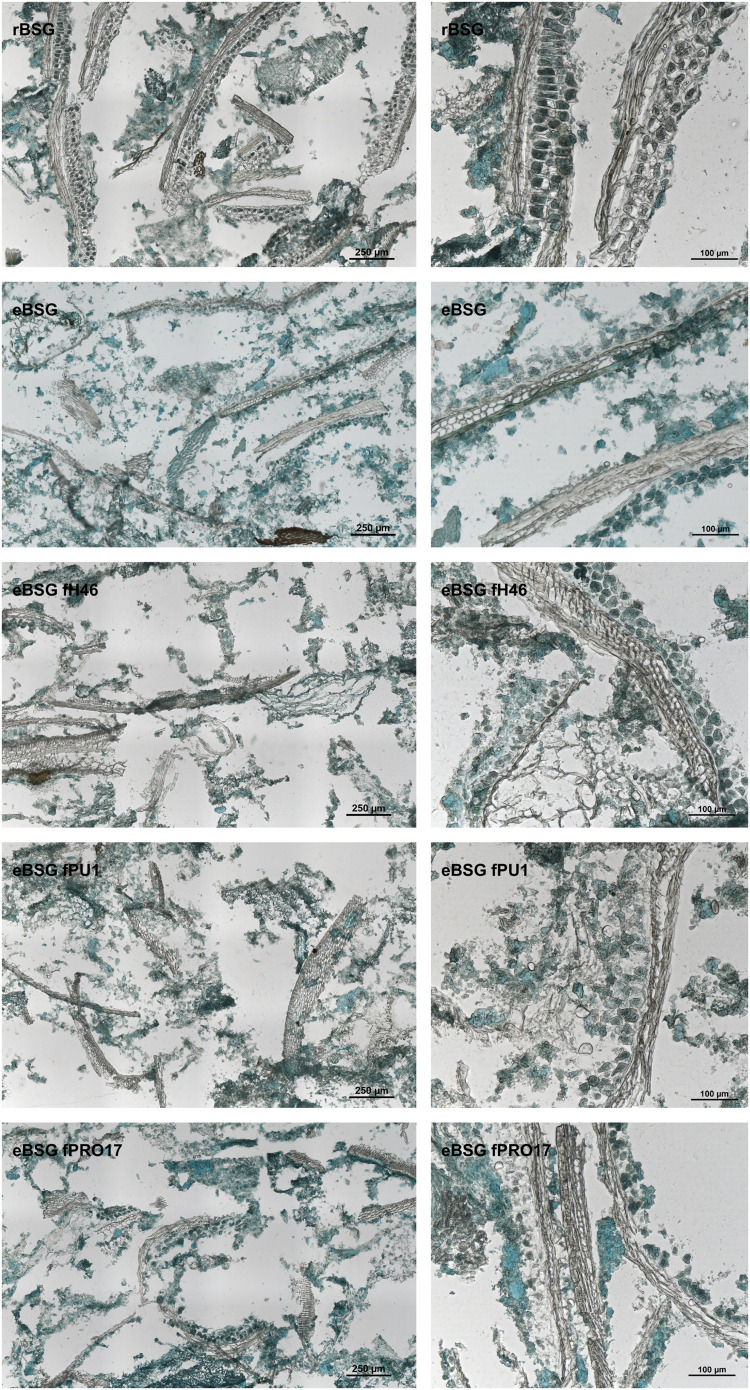
Micrographs of raw BSG (rBSG); BSG treated with xylanase and not inoculated (eBSG); BSG treated with xylanase and fermented with *L. plantarum* PU1 (eBSG fPU1), H46 (eBSG fH46), and PRO17 (eBSG fPRO17). Proteins are shown in green and cell walls/fibers are shown in brown. Scale bars on **left** column are 250 μm and on **right** column are 100 μm.

Intact layers of aleurone cells and large clusters of connective endosperm proteins characterized rBSG. Xylanase treatment (eBSG), caused the extensive degradation of the aleurone cells, and the disassembling of the large protein clusters, apart for some proteins (located in the aleurone cells) still forming globular structures. When fermentation with selected starters followed the enzymatic treatment, no further distinct impact on the microstructure was observed, although the protein appeared unevenly dispersed (Figure 4).

#### Localization of AX

**FIGURE 5:**
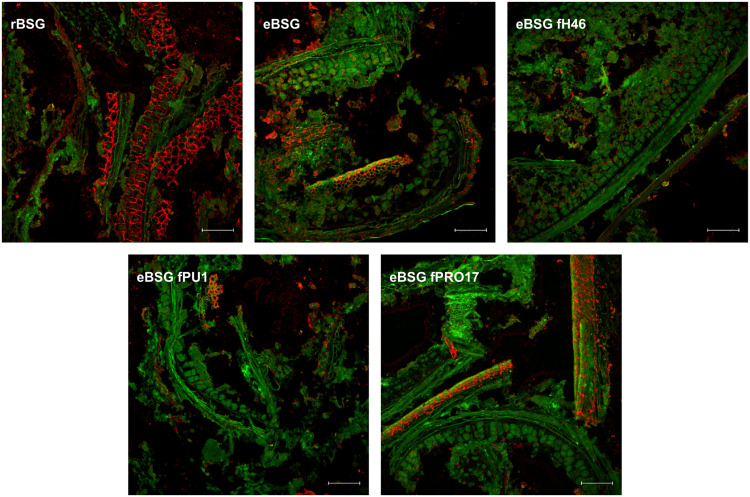
CLSM micrographs of raw BSG (rBSG); BSG treated with xylanase and not inoculated (eBSG); BSG treated with xylanase and fermented with *L. plantarum* PU1 (eBSG fPU1), H46 (eBSG fH46), and PRO17 (eBSG fPRO17). Arabinoxylans are shown in red after labeling with LM11 antibody. BSG morphology is shown in green with autofluorescence. Scale bars correspond to 25 μm.

In rBSG, AX were mainly located in the aleurone cell walls. Small amounts were also observed in the pericarp. In eBSG, as expected, AX of the aleurone cells walls were markedly degraded and disorganized. Contrarily to rBSG, AX fragments dispersed in the matrix were observed in eBSG. Very few AX were detectable in bioprocessed samples inoculated with LAB after the xylanase treatment, both in aleurone cell walls and dispersed in the matrix, especially when *L. plantarum* PU1 and PRO17 were used as starters. Nevertheless, residual bound AX were seen in pericarp after enzymatic treatment and fermentation. Overall, the sequential bioprocessing treatments caused the protein arrangement in rows with globular structures (Figure 5).

#### Dietary Fibers Analysis

Compared to rBSG, all bioprocessed BSG also had a lower content of insoluble fibers (below 40% against the 49 ± 4% of rBSG) and higher content of soluble fibers (ranging from 4.7 ± 1.9 to 5.6 ± 0.7% against 1.5 ± 0.3% found in rBSG).

### *Ex vivo* Assays on Human Keratinocytes

Brewers’ spent grain antioxidant properties were investigated on human keratinocytes line NCTC 2544. The cells subjected to oxidative stress were grown in the presence of untreated and bioprocessed BSG. Compared to cell viability after oxidative stress (25.5 ± 3.6%), α-tocopherol, used as reference for the antioxidant activity, significantly (*P* < 0.05) increased cell survival. All the samples had a protective effect comparable to that of α-tocopherol when tested at 0.1 and 0.5 mg/mL (cell survival of ca. 50%). When tested at 1 mg/mL, survival significantly decreased up to 30% in rBSG and eBSG, whereas only fermented BSG showed protection with a vitality of 42.41 ± 3.37, 39.29 ± 4.62, and 43.30 ± 1.18% for eBSG fH46, eBSG fPRO17, and eBSG fPU1, respectively.

### Characterization of the Phenolic Profile

#### Free and Bound Phenolic Compounds

Phenolic and other polar compounds of free and bound extracts were separated and identified by UPLC-PDA-ESI-QTOF. Supplementary Table S1 summarizes the information related to the compounds tentatively identified: retention times, experimental and calculated m/z, molecular formula, fragments, score, and error (ppm). Forty-three phenolic compounds were identified between free and bound profiles (details in Supplementary Material, see section “Characterization of the Phenolic Compounds”).

Bound phenolic profile was characterized by several phenolic acids and their derivatives. In particular, bound phenolics in rBSG resulted more than 50-fold higher than free phenolics (1768.11 ± 61.23 against 33.40 ± 1.19 mg/kg d.m.), this difference drastically changed with the treatment with xylanase, which freed 25% of bound phenols, most of which, ferulic acid derivatives (Table 2). Compared to rBSG, bound phenolics concentration decreased (*P* < 0.05) in eBSG, indeed, decrease of all ferulic acid dimers, trimers and tetramers was observed. Nevertheless, compared to eBSG, ferulic acid oligomers concentrations significantly increased in all fermented samples, up to three times higher, when *L. plantarum* PU1 was used as starter. Ferulic acid dimers were detected in higher concentration than rBSG only when fermentation was carried out by *L. plantarum* PU1 and PRO17. The combined effect of enzyme and fermentation allowed an increase of the total phenols extractability from the matrix, up to 18%, therefore exceeding 2 g/kg.

**TABLE 2:** Quantification (mg/kg d.w.) of the phenolic compounds identified in raw BSG (r-BSG), treated with xylanase (eBSG), treated with xylanase (5 h at 50°C) and sequentially fermented (at 30°C for 24 h) with *L. plantarum* PU1 (eBSG fPU1), H46 (eBSG fH46), and PRO17 (eBSG fPRO17) obtained by HPLC-DAD-ESI-QTOF-MS.

	**rBSG**	**eBSG**	**eBSG fH46**	**eBSG fPU1**	**eBSG fPRO17**
**Bound phenolic compounds**					
5,7-dihydroxychromone	11.360.48^a^	4.300.12^b^	4.650.17^b^	2.930.12^c^	3.080.11^c^
Vanillic acid	6.780.27^c^	8.250.34^b^	12.070.29^a^	11.660.35^a^	12.110.37^a^
Catechin	5.890.26^a^	5.120.20^b^	6.110.18^a^	5.800.23^a^	1.390.10^c^
Caffeic acid	34.981.40^ab^	28.331.10^c^	32.361.17^b^	38.701.23^a^	37.961.28^a^
Epicatechin	1.530.06^b^	1.410.06^b^	1.710.05^a^	1.730.06^a^	1.020.03^c^
*p*-cumaric acid	40.851.78^b^	30.431.21^c^	39.991.26^b^	42.291.36^b^	47.581.68^a^
*o*-cumaric acid	181.006.74^b^	139.135.69^c^	184.706.20^b^	195.896.47^ab^	211.777.28^a^
Ferulic acid	52.822.22^a^	31.601.30^c^	38.091.24^b^	22.630.78^d^	23.510.82^d^
Isoferulic acid	311.7913.08^b^	267.189.98^c^	367.0213.48^a^	373.2814.26^a^	377.1913.74^a^
Ferulic acid dimers	568.0820.81^b^	382.0314.66^c^	588.6920.96^ab^	630.8623.46^a^	620.7923.21^a^
Sinapic acid	15.330.71^b^	15.610.69^b^	22.290.78^a^	22.690.78^a^	22.030.81^a^
Ferulic acid tetramers	449.6115.82^c^	332.2812.62^d^	467.7317.34^c^	580.2620.63^a^	535.6618.89^b^
Quercetin	0.560.01^a^	0.440.01^b^	0.520.02^ab^	0.530.01^ab^	0.520.02^ab^
Ferulic acid trimers	87.523.68^d^	59.172.43^e^	111.553.45^c^	141.034.57^a^	121.624.24^b^
Total	1768.1161.23^c^	1305.2953.51^d^	1877.4851.33^b^	2070.2879.74^a^	2016.2177.64^a^
**Free phenolic compounds**					
Dihydroferulic acid	3.350.11^b^	3.410.14^b^	52.791.78^a^	51.511.65^a^	48.371.59^a^
Dihydrocaffeic acid	5.090.21^b^	5.630.22^a^	36.611.23^c^	31.481.46^d^	33.561.31^c^
Phloretic acid	23.150.87^c^	22.830.84^c^	69.302.39^a^	56.092.31^b^	58.442.12^b^
Ferulic acid derivatives	−	444.7918.20^a^	316.6211.98^b^	294.4710.97^b^	313.6911.23^b^
Ferulic acid	−	1.280.05^c^	4.390.12^a^	3.390.10^b^	3.420.11^b^
Chrysoeriol	0.890.04^a^	0.890.03^a^	0.850.02^a^	0.790.02^a^	0.840.03^a^
Xanthohumol	0.930.03^a^	0.860.02^ab^	0.800.01^ab^	0.730.02^b^	0.770.03^b^
Total	33.401.19^c^	479.6917.71^a^	481.3616.58^a^	438.4715.18^b^	459.0916.84^ab^

*The data are the means of three independent experiments ± standard deviations (*n* = 3). ^*a*–*e*^Values in the same row, with different superscript letters, differ significantly (*P* < 0.05).*

A slight but significant (*P* < 0.05) decrease was observed for quercetin in eBSG compared to rBSG. As for catechin and epicatechin, a significant decrease was also observed during fermentation with *L. plantarum* PRO17. An intense metabolic activity on phenolic acids was observed. Indeed, higher concentrations of vanillic, caffeic, *o*-coumaric, and sinapic acid, and lower of ferulic acid were found in the bound fraction of the fermented BSG.

Free phenolics profile of rBSG was less complex than bioprocessed BSG (Table 2). Ferulic acid derivatives were not found in rBSG instead they were at concentration higher than 400 mg/kg in eBSG. A slight but significant reduction of their content was observed after fermentation, probably due to a further release of ferulic acid and its consequent reduction to dihydroferulic acid, which increased up to 15-fold during fermentation. Caffeic and coumaric acid liberated from bound phenolics during the incubation were also metabolized to their respective reduced forms, dihydrocaffeic and phloretic acids which increased up to seven and threefold, respectively. The highest metabolites concentrations were found for eBSG fH46.

#### Free Proanthocyanidin Quantification by HPLC-FLD

Extracts from raw and bioprocessed BSG, containing the proanthocyanidin fraction of all phenolic compounds, were subjected to chromatographic analysis. Although the separation poorly allowed to distinguish among the galloylated forms, a clear separation between monomers and oligomers was obtained. Despite the similar trend among samples, proanthocyanidin monomers resulted almost 10-fold higher compared to the data of free phenolics, reaching 68 ± 3 mg/kg in rBSG. Indeed, fluorescence was proved to be more suitable than UV detection, with increased selectivity for procyanidins and reduced interferences from other absorbing compounds. Multiple studies found the detection limits using fluorescence to be nearly one thousand times lower than those observed with UV detection (Hümmer and Schreier, 2008).

When BSG was fermented after the enzymatic treatment, polymeric forms of proanthocyanidins were under the detection limit (Supplementary Figure S1). A slight but significant reduction of proanthocyanidin oligomers was also found in eBSG (44 ± 3 against 32 ± 2 mg/kg of rBSG), probably due to the activity of endogenous microbiota.

### Characterization of Potentially Bioactive Peptides

Antioxidant activity of the WSE of bioprocessed BSG was not affected (*P* < 0.05) by enzymatic digestion and heating. All the fractions obtained from the first separation (corresponding to the permeate at cut-off 50, 30, 10, and 3 kDa) did not show decrease in radical scavenging activity. Based on these results, it was hypothesized that active compounds possessed molecular mass lower than 3 kDa. WSE permeate at <3 kDa was further purified by RP-FPLC, obtaining 33 fractions (peptide concentration in the range 0.40 ± 0.02–12.05 ± 0.06 mg/mL).

The highest antioxidant activity was found in fractions 11 and 15 (31 and 39%, respectively), while only weak activity (19–22%) was found in fractions 2 and 22. Fractions eluted at 12 and 20% of eluent B. Fractions were characterized by peptide concentration ranging from 5.11 ± 0.05 to 8.05 ± 0.06 mg/mL).

Five peptides, having 8–10 amino acid residues, were identified by nano-LC-ESI-MS/MS analysis. A mixture of peptides was identified in all the active fractions. LFGFTYLR (molecular mass of 1016.21 Da), LVLANAIYFK (1151.410 Da), and VGYVANFCK (1000.18 Da) had net charge of 1. While IFLENVIR (1003.21 Da) and EVQMDFVR (1000.18 Da) had net charge of 0 and −1, respectively. Peptides were reported in the NCBI database as encrypted into sequences of *H. vulgare* proteins: beta-amylase (LFGFTYLR), alpha-amylase/trypsin inhibitor (EVQMDFVR), and predicted protein (VGYVANFCK); *Z. mays* proteins: histone H4 (IFLENVIR) and putative serpin-Z12 (LVLANAIYFK). Identified peptides contained from 8 to 10 amino acid residues. Total hydrophobicity ratio ranged from 50 (LFGFTYLR and EVQMDFVR) to 70% (LVLANAIYFK). Peptides were rich in tyrosine (Y), leucine (L), alanine (A), isoleucine (I), valine (V), phenylalanine (F), and contained one or more aromatic amino acids.

## Discussion

The increasing awareness of the benefits of a healthier diet and lifestyle is encouraging the study and management of antioxidant compounds in food, since they represent the most feasible way of protection against free radicals. In humans, the generation of free radicals, unstable and highly reactive chemical species, increases as consequence of the exposure to different physiochemical conditions and pathological states. Free radicals are responsible for oxidative stress, associated to aging, cancer, cardiovascular and neurodegenerative diseases, atherosclerosis, and inflammatory state (Lobo et al., 2010).

In this study, BSG was bioprocessed with the aim of enhancing its antioxidant potential, foreseeing its use as functional food ingredient. The potential of food fermentation in improving antioxidant properties has been highlighted in multiple occasions (for review see Verni et al., 2019). Thanks to its high content of phenolic acids and other potentially bioactive precursors, BSG is a good substrate for the formation of compounds with high antioxidant potential. A preliminary screening of thirty-tree LAB (details in Supplementary Material) allowed the selection of three strains responsible for the most relevant increase of the antioxidant activity in the fermented matrix. Hence, *L. plantarum* PU1, H46 and PRO17, were selected and used for the set-up of a combined bioprocess including the use of a food-grade xylanase.

Together with the increase of the radical scavenging activity, tested on DPPH and ABTS radicals, the capacity of the strains to release phenolic compounds and protein derivatives (peptides and free amino acids) was investigated, since all these compounds are potentially responsible for the antioxidant properties of a fermented matrix (Verni et al., 2019). In addition to fermentation, xylanase was used to degrade the hemicellulose fraction (Robertson et al., 2010), therefore improving the release of phenolic compounds bound to AX of the BSG cell walls. The enzymatic pre-treatment at 50°C for 5 h markedly increased the radical scavenging activity compared to the simultaneous enzymatic/fermentative bioprocess and was therefore selected as the optimal bioprocessing option.

Bioprocessed BSG, subjected to the sequential enzymatic/fermentative treatment, was also able to inhibit lipid peroxidation during 8 days of incubation. Among the different factors involved in this effect, phenolic compounds might have played a crucial role, since they are able to scavenge lipid-derived radicals thereby breaking the free radical chain reaction of lipid peroxidation (Cheng et al., 2003). From a technological point of view, the persistence of the antioxidant activity could prevent food from oxidation, thus reducing loss of nutrients, and maintaining texture, color pigments, taste, freshness, functionality, and aroma (Verni et al., 2019).

To clarify the effects of bioprocessing on BSG, the overall morphology of samples was examined with light microscopy and both content and localization of AX, using differential staining, were visualized by CLSM. As expected, the xylanase treatment allowed an extensive cell wall disruption, contributing to the release of bound molecules and pouring compounds trapped in the cellular compartments to the matrix, becoming available to the LAB catabolic activity during the following step of fermentation.

Since *in vitro* assays are only considered as predictive tools for the antioxidant activity *in vivo* and testing a substance directly on animals or human is not an easy approach, different methods comprising cellular models were recently developed (Verni et al., 2019). In this study, a well-known human keratinocyte cell line was subjected to oxidative-induced stress with peroxide hydroxide and subjected to the MTT assay, which allows the cell survival estimation after stress exposure. The test was performed in presence of the extracts obtained from treated and untreated BSG, aiming at evaluating their protective effect against oxidative stress. Compared to the untreated BSG, bioprocessing conferred to the matrix a relevant protective activity in all the tested conditions, especially when *L. plantarum* PU1 was used as starter. Different BSG phenolic acids (e.g., ferulic, *p*-coumaric, sinapic, and caffeic acids) were previously correlated to protection against DNA oxidative damage (McCarthy et al., 2012). Moreover, previous studies already correlated the presence of bioactive peptides and phenolic compounds generated during LAB fermentation with the improved cell survival to the oxidative stress (Verni et al., 2019). Based on these considerations, phenol, and peptide profiles of bioprocessed BSG were characterized.

Bound phenolic profile of both raw and bioprocessed BSG was mainly characterized by phenolic acids and their derivatives. Several ferulic acid derivatives were found, and their concentration was higher in bioprocessed BSG, in which the extensive degradation of the cell walls caused the increase of the total phenols extractability. Catechin, epicatechin, quercetin, and safrole, this latter previously identified in hop (Yan et al., 2018), were found in both free and bound phenols fraction. During the industrial brewing process, the trub separated after boiling the wort is also added to BSG, therefore, it is possible to find hop components.

A relevant amount of the bound phenolics in untreated BSG, mainly represented by ferulic acid derivatives, was liberated as the consequence of xylanase treatment. Being xylanase the main enzymatic activity found in Depol 761P, it is hypothesized that the increase of phenolic compounds can be ascribed to direct and indirect effect of the fibers hydrolysis, as confirmed by the microstructure analysis reported above.

The free phenolics profile was characterized by the products of microbial metabolism of phenolic acids and their derivatives especially in eBSG and fermented BSG. Dihydroferulic, dihydrocaffeic, and phloretic acids are, in fact, the products of ferulic, caffeic, and coumaric acid reduction, respectively (Filannino et al., 2014). Overall, the highest metabolites concentration was found in eBSG fH46.

Results of this study revealed the complete absence of ferulic acid and its derivatives in untreated BSG, and their high content in eBSG. When fermentation followed enzymatic treatment, a decrease of the ferulic acid and its derivatives was observed as consequence of the conversion in dihydroferulic acid. Feruloyl esterases release ferulic acid and other cinnamic acids and many LAB, especially of the genus *Lactobacillus*, have been reported to possess such activity. Hydroxybenzoic and hydroxycinnamic acids, whose high concentrations negatively affect the microbial physiological functions (Gänzle, 2014), may be also decarboxylated by LAB to the corresponding phenol or vinyl derivatives or hydrogenated by phenolic acid reductases, and their products can exert higher biological activities than the precursors (Gänzle, 2014).

To complete the phenolics characterization, free proanthocyanidins were selectively extracted and quantified. Although their total content was lower than that found in a recent study on BSG (Martín-García et al., 2019), the amount of monomers resulted markedly higher than that previously found, most likely because this BSG contained maize, which was reported to have a high content of these forms (up to 43 g/kg) (Chen et al., 2017). Combined bioprocessing caused a relevant degradation of the polymeric forms of proanthocyanidins, although monomeric forms did not increase, suggesting a rapid degradation after their release. Lactobacilli and bifidobacteria were indeed previously shown to cleave the heterocyclic ring of catechin and epicatechin (Saìnchez-Pataìn et al., 2012).

In addition to phenolic compounds, BSG has been recently reported as a potential source of antioxidant peptides, released through enzymatic hydrolysis with proteases (Cermeño et al., 2019). In this study, since WSE was characterized by an increase of antioxidant activity and high peptide concentration, a role of the LAB in the release of active sequences through proteolysis was hypothesized. In particular, the activity was not affected by enzymatic digestion and heating, thus confirming the stability of the active sequences. Indeed, to be effective, antioxidant peptides should have overcome hydrolysis and modifications at the intestinal level and reach their targets (Sarmadi and Ismail, 2010).

Mixtures of small peptides, not reported before as antioxidant sequences, were identified in the active and purified WSE fractions obtained from eBSG fPU1. Overall, it was hypothesized that the strongest antioxidant activity was ascribed to the synergic effect rather than to the individual activity of the single peptides (Coda et al., 2012). All the sequences resulted encrypted in native barley and maize native proteins. As previously reported as common feature of the antioxidant sequences (Zou et al., 2016), the five identified peptides contained from 8 to 10 amino acid residues. Total hydrophobicity ratio ranged from 50 to 70%. This is an important feature, since hydrophobic amino acids enhance the solubility of peptides in lipids, thus facilitating access to hydrophobic radical species and to hydrophobic PUFAs (polyunsaturated fatty acids) (Sarmadi and Ismail, 2010; Zou et al., 2016). Identified peptides were rich in hydrophobic amino acids which are frequently included in the antioxidant peptides structure (Zou et al., 2016). Moreover, all identified sequences also contained one or more aromatic amino acids, capable to donate protons to electron-deficient radicals, therefore enhancing radical-scavenging activity of the molecule (Sarmadi and Ismail, 2010).

Despite being rich in fiber, proteins and phenolic compounds, BSG is still underutilized for human nutrition, mostly due to its characteristics (i.e., high fiber content and instability) which make BSG a challenging material for food applications. Different studies already described the positive role of pre-treatments with xylanases or fermentation on sensory, technological, and rheological properties of BSG-based foods (Waters et al., 2012; Ktenioudaki et al., 2015). Thus, new biotechnological approaches to improve BSG functionality and utilization are needed to reintroduce it in the food chain.

This study showed how tailored bioprocessing, combining a xylanase treatment and a sequential fermentation with selected LAB, could enhance BSG antioxidant potential and showed protective effect against oxidative stress in human keratinocytes. All the bioactive compounds, to exert their functionality, must be absorbed in the gastro-intestinal tract. In this perspective, fermentation was already recognized as an effective way to increase the bioaccessibility of polyphenols in cereal food matrices, promoting their bioavailability at gut level (Ribas-Agustí et al., 2018). Moreover, the absorption of small-molecular weight compound such as phenolic acids is easier compared to larger polyphenols such as proanthocyanidins, which need to be degraded into monomer or dimer units before being absorbed (Carbonell-Capella et al., 2014).

In our work, the bioprocessing with xylanase and LAB fermentation allowed the release of phenolic compounds otherwise bound to the BSG matrix and unavailable for physiological functions. In particular, the results of this investigation highlighted the fundamental role of the starters in releasing specific phenolic compounds and bioactive peptides, thus maximizing the antioxidant effect. However, more studies are required to confirm this functionality *in vivo*.

Brewers’ spent grain with antioxidant properties can be used as novel ingredient for the production of cereal-based, daily-consumed food (i.e., baked goods, pasta), thus helping to increase the intake of antioxidant compounds. In conclusion, the innovative bioprocessing protocol of this study highlights the potential of simple processes as technological option to convert underutilized side streams like BSG into added-value, potential ingredient for innovative food applications.

## Data Availability Statement

The datasets generated for this study are available on request to the corresponding author.

## Author Contributions

MV optimized the bioprocessing protocol, performed the *in vitro* experiments, analyzed the data, and drafted the original manuscript. EP performed the peptide characterization and related data elaboration. AK and SJ performed the microstructure analysis. DP and FR were involved in the *ex vivo* experiments. VV and ED-C oversaw MV in the phenolic profile characterization. RC and CR conceived and designed the experimental plan. CR oversaw the writing process. All authors read and approved the final manuscript.

## Conflict of Interest

DP and FR are employed by Giuliani S.p.A. The remaining authors declare that the research was conducted in the absence of any commercial or financial relationships that could be construed as a potential conflict of interest.

## Abbreviations

ABTS: 2,2 ′ -azino-di-(3-ethylbenzthiazoline sulfonate)
AX: arabinoxylans
BHT: butylated hydroxytoluene
BSA: bovine serum albumin
BSG: brewers spent grain
CLSM: confocal laser scanning microscopy
DPPH: 2,2-diphenyl-1-picrylhydrazyl
eBSG: enzyme treated brewers spent grain
eBSG fH46: enzyme treated brewers spent grain fermented with *L. plantarum* H46
eBSG fPRO17: enzyme treated brewers spent grain fermented with *L. plantarum* PRO17
eBSG fPU1: enzyme treated brewers spent grain fermented with *L. plantarum* PU1
EF30: bioprocessing protocol including simultaneous enzymatic treatment and fermentation at 30 ° C for 24 h
E50 + F30: Bioprocessing protocol including sequential enzymatic treatment at 50 ° C for 5 h and fermentation at 30 ° C for 24 h
FBS: fetal bovine serum
HPLC-FLD: high-performance liquid chromatography with fluorescence detection
LAB: lactic acid bacteria
ME: methanolic extracts
MRS, De Man: Rogosa and Sharpe
MMT: 3-(4,5-dimethylthiazol-2-yl)-2,5-diphenyltetrazolium bromide
OPA: *o*-phtaldialdehyde
PBS: phosphate-buffered saline
PUFAs: polyunsaturated fatty acids
rBSG: raw BSG
RP-FPLC: reversed-phase fast performance liquid chromatography
TFAA: total free amino acids
UPLC-PDA-ESI-QTOF: ultra-performance liquid chromatography with photodiode array detection electrospray ionization quadrupole and time-of-flight mass spectrometry
WSE: water/salt soluble extracts.
