# Supplementary material for: Bioprocessing of Brewers’ Spent Grain Enhances Its Antioxidant Activity: Characterization of Phenolic Compounds and Bioactive Peptides
Source: Front Microbiol. 2020 Jul 31;11:1831. doi: 10.3389/fmicb.2020.01831 (PMC7411387; doi:10.3389/fmicb.2020.01831)
Supplement: Supplementary file 1 [file Data_Sheet_1.docx]

Supplementary Material

**S1 Characterization of the phenolic compounds**

S1.1 Extraction of phenolic compounds and UPLC-PDA-ESI-QTOF analysis

Free and bound phenolics were extracted as described in Verardo et al. (2011). The analysis of BSG free and bound polyphenols was carried out with the use of an ACQUITY Ultra Performance LC system equipped with photodiode array detector with a binary solvent manager (Waters Corporation, Milford, MA, USA) series with a mass detector Q/TOF micro mass spectrometer (Waters) equipped with an electrospray ionization (ESI) source operating in negative mode at the following conditions: capillary voltage, 2300 kV; source temperature, 100°C; cone gas flow, 40 L/Hr; desolvatation temperature, 500°C; desolvatation gas flow, 11000 L/Hr; and scan range, m/z 50–1500. Separations of individual polyphenols were carried out using an ACQUITY UPLC BEH Shield RP18 column (1.7 µm, 2.1 mm × 100 mm; Waters Corporation, Milford, MA, USA) at 40°C. The elution gradient was carried out using water containing 1% acetic acid (A) and acetonitrile (B), and applied as follows: 0 min, 1% B; 2.3 min, 1% B; 4.4 min, 7% B; 8.1 min, 14% B; 12.2 min, 24% B; 16 min, 40% B; 18.3 min, 100% B, 21 min, 100% B; 22.4 min, 1% B; 25 min, 1% B. The sample volume injected was 2 µL and the flow rate used was 0.6 mL/min. The compounds were monitored at 280 nm. Integration and data elaboration were performed using MassLynx 4.1 software (Waters Corporation, USA). For the quantification of phenolic compounds, solutions of ferulic acid, chlorogenic acid, catechin and quercetin in methanol were prepared and used as standard.

S1.2 Identification of free and bound phenolic compounds

Phenolic and other compounds of free and bound extracts were separated and identified by UPLC-PDA-ESI-QTOF. Table S1 summarizes the information related to the compounds tentatively identified: retention times, experimental and calculated m/z, molecular formula, fragments, score and error (ppm). Forty-three phenolic compounds were identified between free and bound profiles.

Bound phenolic profile was characterized by several phenolic acids and their derivatives. Compound 1, showing molecular ion at *m/z* 177, was identified as 5,7-dihydroxychromone, which was previously found in barley tea (Etoh et al., 2004). Three compounds (2, 4, 11) with molecular ion at *m/z* 167, 179, and 223 were identified as vanillic, caffeic and sinapic acids. Two isomers (6 and 7) showing molecular ion at at *m/z* 163 and providing a fragment at *m/z* 119 were identified as *p-* and *o-*cumaric acids. Caffeic and coumaric acids provided fragments at *m/z* 135 and 119, respectively, corresponding to the loss of CO_2_, whereas sinapic acid provided 4 typical fragments at *m/z* 193, 164, 149 and 121. Two other compounds (8 and 9) with molecular ion at *m/z* 193 were identified as ferulic and isoferulic acid as confirmed by coelution with ferulic acid standard and retention times of a previous work (Zengin et al., 2018). All the hydroxycinnamic and hydroxybenzoic acids identified were previously found in barley, maize and BSG as well (Dykes and Rooney, 2007; Mussatto et al., 2007). Catechin and epicatechin (3 and 5) were identified at *m/z* 289 by mass spectra and coelution with a commercial standard, as well as quercetin at *m/z* 301, while compound 13, showing molecular ion at *m/z* 161, was identified as safrole.

Compounds 14, 15, 17 at *m/z* 389 and 10 at *m/z* 385 were identified as ferulic acid dimers based on the fragmentation pattern of the MS^2^ signal, matching that reported by Chandrasekara and Shahidi (2011). Three more isomers (compounds 20, 22 and 24) at *m/z* 387 were identified as ferulic acid dehydrodimers, accordingly to Hernandez et al. (2001), showing fragment ions at *m/z* 341 and 193, corresponding to the loss of CO_2_ and ferulic acid, respectively. Compounds 18 and 19, with *m/z* 403, were identified as tetrahydrofuran ferulic acid dehydrodimers as reported in Pedersen et al. (2015). One more ferulic acid derivative, previously found in maize (Mélida et al., 2010), was identified at *m/z* 401 and providing fragments ions at *m/z* 357 and 313. Two isomers (28 and 29) at *m/z* 577 and providing fragments at *m/z* 385 and 341, respectively, were identified as ferulic acid trimers. Whereas, four isomers (16, 23, 25 and 26) with molecular ion at *m/z* 771, providing the most intense fragment ion at *m/z* 341, were identified as dehydrotetraferulic acids (Bunzel et al., 2006). One more ferulic acid tetramer was tentatively identified at *m/z* 775, providing fragments at m/z 387, 193 and 134 in MS^2^ signal.

Compounds 1, 2 and 4 of the free phenolics profile, showing *m/z* at 195, 181 and 165, were identified as dihydroferulic, dihydrocaffeic and phloretic acids, respectively, metabolites of ferulic, caffeic and coumaric acid reduction (Filannino et al., 2014). As for bound phenolics, catechin, ferulic acid and safrole were also identified in the free fraction. Three isomers showing *m/z* at 853 (5, 7, and 9) and 721 (6, 8, and 10), were tentatively identified as ferulic acid derivatives providing, in MS^2^ signal, fragments at m/z 775, 703, 325, 265 193, 175 and 325, 265 193, 175, respectively (Schendel et al., 2015). A compound showing *m/z* 831 was tentatively identified as a flavonoid glycoside based on MS^2^ signal. It provided 4 fragments, at *m/z* 741 [M – H - 90]^-^, typical loss of C-linked hexose (Singh et al., 2015), 579 [(M – H) – 90 - 162]^-^, corresponding to the loss of a glucose residue (Pikulski and Brodbelt, 2003), 423 [(M – H) – 90 – 162 - 156]^-^, and 267 [(M – H) – 90 – 162 – 156 - 156]^-^, the 156 loss has been reported as rhamnosyl moiety (Benayad et al., 2014). A similar fragmentation pattern was found for three more compounds at *m/z* 771 and 715 providing the fragments 549, 393, 237 and 165 in MS^2^ signal. Fragmentation of flavonoid at *m/z* 237 was observed by Fabre et al. (2001) for 7-hydroxy flavone [M-H-72]^-^ supported by the loss of CO_2_ and CO. However, their identification was not successful.

Peaks 13 and 14 with molecular ion at *m/z* 299 and 353, were identified as chrysoeriol and xanthohumol, previously found in barley and hop, respectively (Dykes and Rooney, 2007; Kao, 2018). Whereas a compound showing *m/z* 433 and providing fragments at m/z 299, 285, 229 and 131 in MS^2^ signal was tentatively identified as chrysoeriol derivative.

**References**

Benayad, Z., Gómez-Cordovés, C., Es-Safi, N. (2014). Characterization of flavonoid glycosides from fenugreek (*Trigonella foenum-graecum*) crude seeds by HPLC–DAD–ESI/MS analysis. Int. J. Mol. Sci. 15:20668-20685. [doi: 10.3390/ijms151120668](https://doi.org/10.3390/ijms151120668)

Bunzel, M., Ralph, J., Brüning, P., Steinhart, H. (2006). Structural identification of dehydrotriferulic and dehydrotetraferulic acids isolated from insoluble maize bran fiber. J. Agric. Food Chem. 54:6409-6418. [doi: 10.1021/jf061196a](https://doi.org/10.1021/jf061196a)

Chandrasekara, A., Shahidi, F. (2011). Determination of antioxidant activity in free and hydrolyzed fractions of millet grains and characterization of their phenolic profiles by HPLC-DAD-ESI-MSn. J. Funct. Foods 3:144-158. [doi: 10.1016/j.jff.2011.03.007](https://doi.org/10.1016/j.jff.2011.03.007)

Dykes, L., Rooney, L.W. (2007). Phenolic compounds in cereal grains and their health benefits. Cereal Foods World 52:105-111.

Etoh, H., Murakami, K., Yogoh, T., Ishikawa, H., Fukuyama, Y., Tanaka, H. (2004). Anti-oxidative compounds in barley tea. Biosci. Biotech. Biochem. 68: 2616-2618. [doi: 10.1271/bbb.68.2616](https://doi.org/10.1271/bbb.68.2616)

Fabre, N., Rustan, I., de Hoffmann, E., Quetin-Leclercq, J. (2001). Determination of flavone, flavonol, and flavanone aglycones by negative ion liquid chromatography electrospray ion trap mass spectrometry. J. Am. Soc. Mass Spec. 12:707-715. [doi: 10.1016/S1044-0305(01)00226-4](https://doi.org/10.1016/S1044-0305(01)00226-4)

Filannino, P., Gobbetti, M., De Angelis, M., Di Cagno, R. (2014). Hydroxycinnamic acids used as external acceptors of electrons: an energetic advantage for strictly heterofermentative lactic acid bacteria. Appl. Environ. Microb. 80:7574-7582. [doi: 10.1128/AEM.02413-14](https://doi.org/10.1128/AEM.02413-14)

Hernanz, D., Nuñez, V., Sancho, A. I., Faulds, C.B., Williamson, G., Bartolomé, B., et al. (2001). Hydroxycinnamic acids and ferulic acid dehydrodimers in barley and processed barley. J. Agric. Food Chem. 49:4884-4888. [doi: 10.1021/jf010530u](https://doi.org/10.1021/jf010530u)

Kao, T.H. (2018). Health Potential for Beer Brewing Byproducts. In Current Topics on Superfoods. In K. College (Ed.), Current Topics on Superfoods (pp. 49-66) IntechOpen.

Mélida, H., García-Angulo, P., Alonso-Simón, A., Álvarez, J. M., Acebes, J. L., Encina, A. (2010). The phenolic profile of maize primary cell wall changes in cellulose-deficient cell cultures. Phytochemistry 71:1684-1689. [doi: 10.1016/j.phytochem.2010.06.013](https://doi.org/10.1016/j.phytochem.2010.06.013)

Mussatto, S.I., Dragone, G., Roberto, I.C. (2006). Brewers' spent grain: generation, characteristics and potential applications. J. Cereal Sci. 43:1-14. [doi: 10.1016/j.jcs.2005.06.001](https://doi.org/10.1016/j.jcs.2005.06.001)

Mussatto, S.I., Dragone, G., Roberto, I.C. (2007). Ferulic and *p*-coumaric acids extraction by alkaline hydrolysis of brewer's spent grain. Ind. Crops Prod. 25:*2*31-237. [doi: 10.1016/j.indcrop.2006.11.001](https://doi.org/10.1016/j.indcrop.2006.11.001)

Pedersen, M.B., Bunzel, M., Schäfer, J., Knudsen, K.E.B., Sørensen, J. F., Yu, S., et al. (2015). Ferulic acid dehydrodimer and dehydrotrimer profiles of distiller’s dried grains with solubles from different cereal species. J. Agric. Food Chem. 63:2006-2012. [doi: 10.1021/jf505150g](https://doi.org/10.1021/jf505150g)

Pikulski, M., Brodbelt, J.S. (2003). Differentiation of flavonoid glycoside isomers by using metal complexation and electrospray ionization mass spectrometry. J. Am. Soc. Mass Spec. 14:1437-1453. [doi: 10.1016/j.jasms.2003.07.002](https://doi.org/10.1016/j.jasms.2003.07.002)

Rao, S., Santhakumar, A.B., Chinkwo, K.A., Blanchard, C.L. (2018). Q-TOF LC/MS identification and UHPLC-Online ABTS antioxidant activity guided mapping of barley polyphenols. Food Chem. 266:323-328. [doi: 10.1016/j.foodchem.2018.06.011](https://doi.org/10.1016/j.foodchem.2018.06.011)

Schendel, R.R., Becker, A., Tyl, C.E., Bunzel, M. (2015). Isolation and characterization of feruloylated arabinoxylan oligosaccharides from the perennial cereal grain intermediate wheat grass (*Thinopyrum intermedium*). Carbohydr. Res. 407:16-25. [doi: 10.1016/j.carres.2015.01.006](https://doi.org/10.1016/j.carres.2015.01.006)

Singh, A., Kumar, S., Bajpai, V., Reddy, T.J., Rameshkumar, K.B., Kumar, B. (2015). Structural characterization of flavonoid C‐and O‐glycosides in an extract of *Adhatoda vasica* leaves by liquid chromatography with quadrupole time‐of‐flight mass spectrometry. Rapid Commun. Mass Spectrom. 29:1095-1106. [doi: 10.1002/rcm.7202](https://doi.org/10.1002/rcm.7202)

Verardo, V., Arráez-Román, D., Segura-Carretero, A., Marconi, E., Fernández-Gutiérrez, A., Caboni, M.F. (2011). Determination of free and bound phenolic compounds in buckwheat spaghetti by RP-HPLC-ESI-TOF-MS: Effect of thermal processing from farm to fork. J. Agric. Food Chem. 59:7700-7707. [doi: 10.1021/jf201069k](https://doi.org/10.1021/jf201069k)

Zengin, G., Uysal, A., Diuzheva, A., Gunes, E., Jekő, J., Cziáky, Z., et al. (2018). Characterization of phytochemical components of *Ferula halophila* extracts using HPLC-MS/MS and their pharmacological potentials: a multi-functional insight. J. Pharmaceut. Biomed. 160:374-382. [doi: 10.1016/j.jpba.2018.08.020](https://doi.org/10.1016/j.jpba.2018.08.020)

**Table S1.** Identification of free and bound compounds of brewers’ spent grain raw and bioprocessed with xylanase and/or selected lactic acid bacteria by HPLC-DAD-ESI-QTOF-MS.

| **No.** | **Polar compound** | **RT (min)** | **Molecular Formula** | ***m/z* exp** | ***m/z* calc** | **Error (ppm)** | **Score (%)** | **Fragments (MS^2^)** | **References** |
| --- | --- | --- | --- | --- | --- | --- | --- | --- | --- |
| ***Bound phenolic compounds*** | |  |  |  |  |  |  |  |  |
| **1** | 5,​7-​Dihydroxychromone | 4.841 | C_9_H_6_O_4_ | 177.0199 | 177.0188 | 6.2 | 86.20 |  | Etoh et al., 2004 |
| **2** | Vanillic acid | 4.974 | C_8_H_8_O_4_ | 167.0352 | 167.0344 | 4.8 | 85.42 |  | Mussatto et al., 2007 |
| **3** | Catechin | 5.209 | C_15_H_14_O_6_ | 289.0723 | 289.0712 | 3.8 | 99.97 |  | Dykes and Rooney, 2007 |
| **4** | Caffeic acid | 5.565 | C_9_H_8_O_4_ | 179.0354 | 179.0344 | 5.6 | 99.97 | 135.0453 | Mussatto et al., 2007 |
| **5** | Epicatechin | 6.409 | C_15_H_14_O_6_ | 289.0715 | 289.0712 | 1.0 | 99.97 |  | Dykes and Rooney, 2007 |
| **6** | *p*-Cumaric acid | 6.417 | C_9_H_8_O_3_ | 163.0401 | 163.0395 | 3.7 | 92.90 | 119.0503 | Mussatto et al., 2007 |
| **7** | *o*-Cumaric acid | 7.546 | C_9_H_8_O_3_ | 163.0403 | 163.0395 | 4.9 | 92.90 | 119.0502 | Mussatto et al., 2007 |
| **8** | Ferulic acid | 7.559 | C_10_H_10_O_4_ | 193.0512 | 193.0501 | 5.7 | 94.83 |  | Mussatto et al., 2007 |
| **9** | Isoferulic acid | 8.233 | C_10_H_10_O_4_ | 193.0507 | 193.0501 | 3.1 | 98.39 |  | Mussatto et al., 2007 |
| **10** | Ferulic acid derivative (cyclodimer) | 8.449 | C_20_H_20_O_8_ | 387.1075 | 387.1080 | -1.3 | 99.64 | 193.0503, 134.0369 | Chandrasekara et al., 2011 |
| **11** | Sinapic acid | 8.510 | C_11_H_12_O_5_ | 223.0616 | 223.0606 | 4.5 | 99.73 | 193.0305, 164.0468. 149.0289, 121.0311 | Dykes and Rooney, 2007 |
| **12** | Ferulic acid derivative (cyclotetramer) | 8.647 | C_40_H_40_O_16_ | 775.2224 | 775.2238 | -1.8 | 84.49 | 387.1075, 193.0503. 134.0369 |  |
| **13** | Safrole | 9.110 | C_10_H_10_O_2_ | 161.0605 | 161.0603 | 1.2 | 91.26 |  | Yan et al., 2018 |
| **14** | Ferulic acid dimer | 9.371 | C_20_H_22_O_8_ | 389.1245 | 389.1236 | 2.3 | 99.88 | 327.1224 |  |
| **15** | Ferulic acid dimer | 9.615 | C_20_H_22_O_8_ | 389.1233 | 389.1236 | -0.8 | 99.91 | 341.1029, 193.0509 |  |
| **16** | Ferulic acid dehydrotetramer | 9.810 | C_40_H_36_O_16_ | 771.1922 | 771.1925 | 0.5 | 82.15 | 341.1031 | Bunzel et al., 2006 |
| **17** | Ferulic acid dimer | 9.884 | C_20_H_22_O_8_ | 389.1232 | 389.1236 | -1.0 | 98.97 | 341.1029, 193.0509 | Bunzel et al., 2006 |
| **18** | 8-8(Tetrahydrofuran)-diferulic acid | 10.045 | C_20_H_20_O_9_ | 403.1035 | 403.1029 | 1.5 | 99.72 |  | Pedersen et al., 2015 |
| **19** | 8-8(Tetrahydrofuran)- diferulic acid | 10.157 | C_20_H_20_O_9_ | 403.1035 | 403.1029 | 1.7 | 85.57 |  | Pedersen et al., 2015 |
| **20** | Ferulic acid dehydrodimer | 11.241 | C_20_H_18_O_8_ | 385.0929 | 385.0923 | 1.8 | 98.80 | 341.1038 |  |
| **21** | Ferulic acid dehydrodimer | 11.464 | C_20_H_18_O_9_ | 401.0879 | 401.0873 | 1.5 | 95.30 | 357.0990, 313.108 | Mélida et al., 2010 |
| **22** | Ferulic acid dehydrodimer | 12.860 | C_20_H_18_O_8_ | 385.0929 | 385.0923 | 1.8 | 98.80 | 341.1038 |  |
| **23** | Ferulic acid dehydrotetramer | 13.289 | C_40_H_36_O_16_ | 771.1922 | 771.1925 | 0.5 | 82.15 | 341.1031 | Bunzel et al., 2006 |
| **24** | Ferulic acid dehydrodimer | 13.380 | C_20_H_18_O_8_ | 385.0929 | 385.0923 | 1.0 | 99.88 |  |  |
| **25** | Ferulic acid dehydrotetramer | 13.550 | C_40_H_36_O_16_ | 771.1929 | 771.1925 | 0.5 | 82.15 | 341.1031 | Bunzel et al., 2006 |
| **26** | Ferulic acid dehydrotetramer | 13.942 | C_40_H_36_O_16_ | 771.1922 | 771.1925 | -0.4 | 97.06 | 485.02, 385.093, 313.108, 193.0508 | Bunzel et al., 2006 |
| **27** | Quercetin | 14.335 | C_15_H_10_O_7_ | 301.0378 | 301.0407 | 10.0 | 74.30 |  | Dykes and Rooney, 2007 |
| **28** | Ferulic acid dehydrotrimer | 14.770 | C_30_H_26_O_12_ | 577.1355 | 577.1346 | 3.3 | 81.12 | 385.0937, 193.0511 | Rao et al., 2018 |
| **29** | Ferulic acid dehydrotrimer | 14.886 | C_30_H_26_O_12_ | 577.1355 | 577.1346 | 1.4 | 74.57 | 341.1044, 193.0503 | Rao et al., 2018 |
|  |  |  |  |  |  |  |  |  |  |
| ***Free phenolic compounds*** | |  |  |  |  |  |  |  |  |
| **1** | Dihydroferulic acid | 2.623 | C_10_H_12_O_4_ | 195.0742 | 195.0736 | 3.46 | 87.98 |  | Filannino et al., 2014 |
| **2** | Dihydrocaffeic acid | 2.905 | C_9_H_10_O_4_ | 181.0508 | 181.0501 | 3.9 | 92.24 | 163.098 | Filannino et al., 2014 |
| **3** | Catechin | 5.3 | C_15_H_14_O_6_ | 289.0723 | 289.0712 | 3.8 | 99.97 |  | Dykes and Rooney, 2007 |
| **4** | Phloretic acid | 5.503 | C_9_H_10_O_3_ | 165.056 | 165.0552 | 4.8 | 86.35 | 147.0446 | Filannino et al., 2014 |
| **5** | Ferulic acid derivative | 6.21 | C_35_H_50_O_24_ | 853.26 | 853.2614 | -1.6 | 99.65 | 775.2266, 703.2077, 325.0923, 265.0711, 193.0511, 175.0428 |  |
| **6** | Ferulic acid derivative | 6.359 | C_30_H_42_O_20_ | 721.2175 | 721.2191 | -2.2 | 99.46 | 325.0923, 265.0711, 193.0511, 175.0428 | Schendel et al., 2015 |
| **7** | Ferulic acid derivative | 6.607 | C_35_H_50_O_24_ | 853.2584 | 853.2614 | -3.5 | 99.96 | 775.2266, 703.2077, 325.0923, 265.0711, 193.0511, 175.0428 |  |
| **8** | Ferulic acid derivative | 6.785 | C_30_H_42_O_20_ | 721.2177 | 721.2191 | -1.9 | 99.95 | 325.0923, 265.0711, 193.0511, 175.0428 | Schendel et al., 2015 |
| **9** | Ferulic acid derivative | 6.913 | C_35_H_50_O_24_ | 853.2587 | 853.2614 | -3.2 | 99.99 | 775.2266, 703.2077, 325.0923, 265.0711, 193.0511, 175.0428 |  |
| **10** | Ferulic acid derivative | 7.009 | C_30_H_42_O_20_ | 721.2183 | 721.2191 | -1.1 | 99.99 | 325.0923, 265.0711, 193.0511, 175.0428 | Schendel et al., 2015 |
| **11** | Ferulic acid | 7.559 | C_10_H_10_O_4_ | 193.0512 | 193.0501 | 5.7 | 95.92 |  | Mussatto et al., 2007 |
| **12** | Safrole | 9.11 | C_10_H_10_O_2_ | 161.0605 | 161.0603 | 1.2 | 95.23 |  | Yan et al., 2018 |
| **13** | Chrysoeriol | 15.612 | C_16_H_12_O_6_ | 299.0529 | 299.0556 | -5.7 | 94.13 | 284.0344 | Kao, 2018 |
| **14** | Xanthohumol | 16.549 | C_21_H_22_O_5_ | 353.1378 | 353.1389 | -3.1 | 66.22 |  | Kao, 2018 |

**Figure S1.** Proanthocyanidin content (mg/kg d.m.) in raw BSG (rBSG), treated with xylanase (eBSG), treated with xylanase and fermented with *Lb. plantarum* PU1 (eBSG fPU1), H46 (eBSG fH46), and *Lb. plantarum* PRO17 (eBSG fPRO17), obtained by HPLC-FLD. The data are the means of three independent experiments ± standard deviations (n=3). Values with different superscript letters, differ significantly (P < 0.05).
